# Simultaneous Multi-Vessel Very Late Stent Thrombosis in Acute ST-Segment Elevation Myocardial Infarction

**DOI:** 10.1155/2021/2658094

**Published:** 2021-12-30

**Authors:** Yuefeng Chen, Michael Amponsah, Cyril Nathaniel

**Affiliations:** Department of Cardiology, Conemaugh Memorial Medical Center, Johnstown, Pennsylvania 15905, USA

## Abstract

Simultaneous multi-vessel very later stent thrombosis (VLST) is a very rare complication of percutaneous coronary intervention (PCI). We present a case of simultaneous multi-vessel VLST as the cause of acute ST-segment elevation myocardial infarction (STEMI). PCI of the culprit vessel was performed at acute presentation. Resolution of in-stent thrombosis in non-culprit vessels was noted on coronary angiography 2 days later. Our case suggests that PCI for culprit lesion in acute setting may be a reasonable option for simultaneous multi-vessel VLST.

## 1. Introductions

VLST is defined as stent thrombosis that occurs after first year after the initial implantation [1]. It is a very rare complication of stent implantation with an incidence of less than 1% during a 4-year follow-up [2]. Simultaneous multi-vessel thrombosis in the setting of acute STEMI has been reported [3], but simultaneous multi-vessel VLST in acute STEMI is extremely rare. Here we report a case of acute STEMI with simultaneous multi-vessel VLST with the last stent implantation of 5 years prior.

## 2. Case Presentation

**Figure 1:**
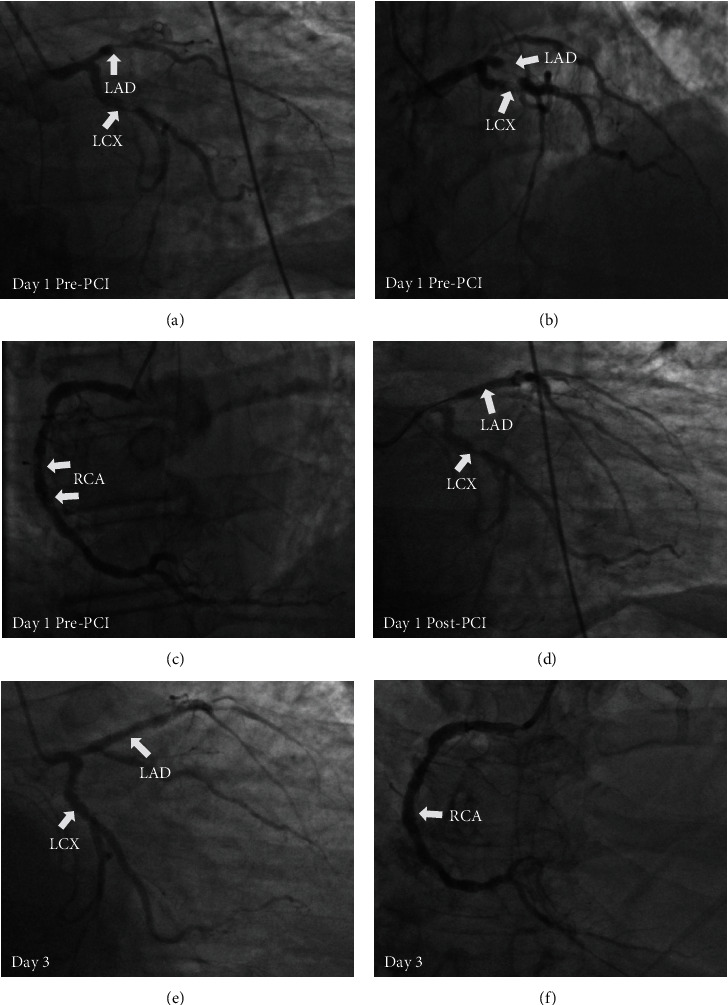
Coronary angiography and LAD primary PCI. Thrombotic occlusion at the site of the proximal LAD stent with TIMI 0 flow (A, B, arrow) and evidence of thrombus at the site of the proximal LCX stent (A, B, arrow) and the mid RCA stent (C, arrows). Following PCI and restoration of TIMI 3 flow in the LAD, there was evidence of remaining thrombus at the site of proximal LCX stent (D, arrow). Resolution of thrombus at the site of the proximal LCX stent (E, arrow) and the mid RCA stent (F, arrow) on day 3 of admission. LAD: left anterior descending artery; LCX: left circumflex artery; RCA: right coronary artery; PCI: percutaneous coronary intervention.

The patient is a 71-year-old male with history of coronary artery disease (CAD) was brought to emergency room for left-sided chest pain for 45 minutes, associated with shortness of breath, diaphoresis and nausea. He has history of PCI of proximal to mid left anterior descending artery (LAD) and proximal left circumflex artery (LCX) with drug-eluting stents (DES) in March, 2012, repeated PCI of mid LAD with DES in March, 2015, and PCI of mid and distal right coronary artery (RCA) in January, 2016. He also has history of hypertension, type II diabetes mellitus and morbid obesity. He was taking Aspirin 325 mg daily, Prasugrel 10 mg daily, Quinapril 40 mg daily, Atorvastatin 80 mg daily. Physical exam revealed body mass index 43.1 kg/m^2^, blood pressure 157/122 mmHg, heart rate 131 beats/minute, respiration rate 24/minute, oxygen saturation 97%, he was in acute respiratory distress, with diaphoresis, tachycardia, tachypnea and bilateral lungs decreased breath sounds, but no wheezing or crackles. Laboratory studies showed SARS-CoV-2 negative, hemoglobin A1C 6.9%, eGFR 60 ml/min, White blood cell 10.8x10^3^/ul, platelet 316x10^3^/ul, B-type natriuretic peptide 340 pg/ml, Troponin I 0.01 ng/ml, Total cholesterol 199 mg/dl, Low-density lipoproteins 140 mg/dl. Initial electrocardiography showed anterior ST segment elevation. He was intubated due to respiratory distress and unable to lie flat. Emergency cardiac catheterization revealed 100% occlusion of proximal to mid LAD with instent thrombosis, and 90% stenosis in proximal LCX and mid RCA stents (Figure 1(a)–1(c)). PCI of proximal to mid LAD was performed and TIMI-3 flow to distal LAD was restored, a Synergy 3.0 x 24 mm DES was then placed and post dilated with a 3.5 mm non-compliant balloon (Figure 1(d) ). The patient was hemodynamically stable and brought back to catheterization laboratory 2 days later for staged PCI of LCX and RCA lesions. However, angiography showed that previously found severe stenosis in the above vessels was no longer visualized (Figure 1(e) and 1(f)), it turned out that previously noted instent stenosis was actually caused by thrombus at the time of acute STEMI and had dissolved spontaneously over time. No PCI of mid LCX and RCA was performed. Patient recovered well and was discharged later.

## 3. Discussions

VLST is an uncommon complication of stent implantation, the incidence is generally less than 1% in newer generation DES [4], and is especially rare after 5 years of initial stenting. In our case, the last stent in the culprit vessel (LAD) was placed 6 years ago, and the stents in LCX and RCA were placed 9 years and 5 years ago, respectively. VLST has been reported to be more often associated with DES than with BMS [5]. It usually presents as acute STEMI, but compared to patients with early and late ST, patients with VLST had better prognosis with lower incidence of major adverse cardiac events and mortality [6–8]. The mechanism of VLST is not fully understood, but is thought to be related to delayed incomplete stent strut endothelialization [9, 10], stent strut malapposition secondary to positive remodeling [10, 11], stent fracture [12], hypersensitivity reaction to polymers [13, 14], chronic inflammation [9, 15], rupture of lipid-laden-like neointima within the DES [10], and discontinuation of antiplatelet therapy [16]. In our case, we did not notice anything from his medical history that known to contribute to VLST, and there was no delay in patient transfer or patient care. Simultaneous triple vessel VLST is extremely rare, only a few cases have been reported [17, 18]. Treatment for multi-vessel ST can be challenging, whether PCI should be performed on all lesions at initial presentation is not certain. In our case, VLST caused 100% occlusion of LAD, while LCX and RCA remained TIMI 3 flow during the procedure, only culprit lesion was treated with PCI initially, the thrombus in the other two vessels dissolved spontaneously in 2 days, suggesting that as long as blood flow is not comprised in non-culprit vessels, culprit lesion only management at presentation may be reasonable. VLST is a multifactorial event and prevention can be difficult, some patients may need oral anticoagulation or long term DAPT therapy. More research needs to be done to identify those who are at high risk for VLST, especially in current era of trending toward short DAPT treatment duration.

## 4. Conclusions

Simultaneous triple vessel VLST is a very rare complication following stent implantation. When blood flow in non-culprit vessels is not comprised, PCI for culprit lesion only at initial presentation may be a reasonable option.

## Data Availability

Data available on request.
